# The Nuclear Envelope as a Regulator of Immune Cell Function

**DOI:** 10.3389/fimmu.2022.840069

**Published:** 2022-06-10

**Authors:** Anna Selezneva, Alasdair J. Gibb, Dean Willis

**Affiliations:** Department of Neuroscience, Physiology & Pharmacology, University College London, London, United Kingdom

**Keywords:** nuclear envelope, nucleus, immune cells, inflammation, lamins, ion channels

## Abstract

The traditional view of the nuclear envelope (NE) was that it represented a relatively inert physical barrier within the cell, whose main purpose was to separate the nucleoplasm from the cytoplasm. However, recent research suggests that this is far from the case, with new and important cellular functions being attributed to this organelle. In this review we describe research suggesting an important contribution of the NE and its constituents in regulating the functions of cells of the innate and adaptive immune system. One of the standout properties of immune cells is their ability to migrate around the body, allowing them to carry out their physiological/pathophysiology cellular role at the appropriate location. This together with the physiological role of the tissue, changes in tissue matrix composition due to disease and aging, and the activation status of the immune cell, all result in immune cells being subjected to different mechanical forces. We report research which suggests that the NE may be an important sensor/transducer of these mechanical signals and propose that the NE is an integrator of both mechanical and chemical signals, allowing the cells of the innate immune system to precisely regulate gene transcription and functionality. By presenting this overview we hope to stimulate the interests of researchers into this often-overlooked organelle and propose it should join the ranks of mitochondria and phagosome, which are important organelles contributing to immune cell function.

## Introduction

The nucleus, one of the defining features of all eukaryotic cells, houses most of the genetic information within the cell and is bounded by the nuclear envelope (NE). In the 1950s research began to elucidate the NE structure and its function (1–3). The prevailing view of this early research was that the NE was just a physical barrier separating nucleoplasm from cytoplasm; however evidence now indicates that this is a too simplistic view of this organelle and that the NE significantly contributes to cellular signalling and regulatory pathways (4–6).

The immune system is an integrated network of organs, cells and biochemical cascades which provides the host with protection against pathogens, noxious exogenous stimuli and trauma. The system also holds memory of pathogen components, allowing it to respond more efficiently when the pathogen is encountered again. While the immune system is required for the continued well-being of an individual, with increased susceptibility to microbial infections and cancers in individuals with supressed immune system; inappropriate activation of the immune system can lead to excessive inflammation, autoimmunity and host tissue destruction. To maintain a balance of appropriate activation, while guarding against self-induced tissue damage/pathology, the immune system employs numerous cellular sensors, regulatory pathways and extra- and intracellular signalling molecules to maintain a controlled appropriate response (7, 8).

Over the last 40 years various organelles, including lysosomes/phagosomes, mitochondria and endoplasmic reticulum (ER), have been shown to be important in regulating the activation and effector functions of both the innate and adaptive immune cells (9). Here, we review the current knowledge regarding the structure and function of NE in cells of the innate and adaptive immune systems and discuss the evidence which suggests the NE is an integrator of both mechanical and chemical signals encountered by immune cells during inflammation.

## Structure and Function of NE

**Figure 1:**
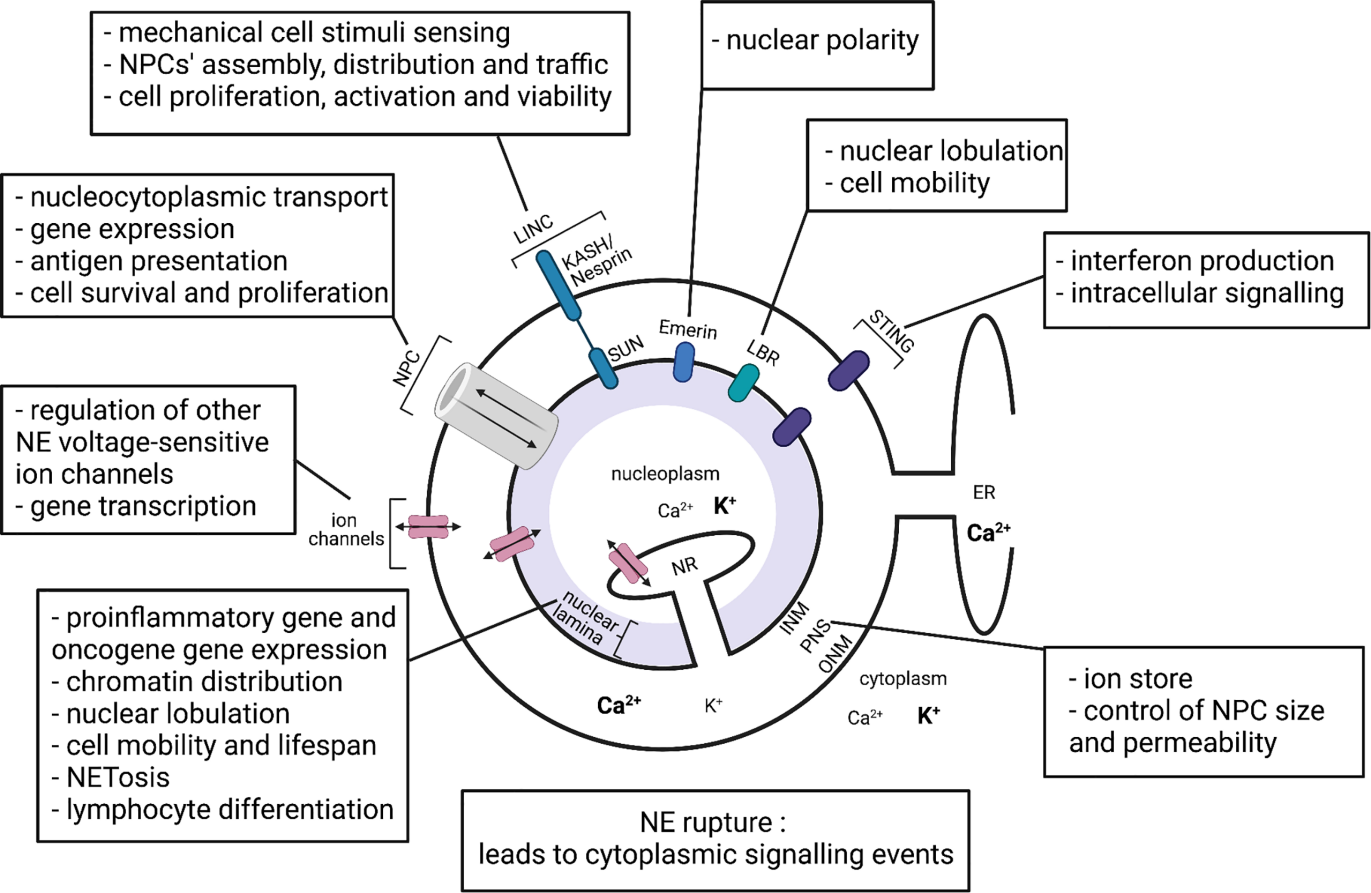
Representation of the nuclear envelope components and their proposed functions in immune cells. INM, inner nuclear membrane; ONM, outer nuclear membrane; PNS, perinuclear space; NR, nucleoplasmic reticulum; ER, endoplasmic reticulum; NPC, nuclear pore complex; LINC, linker of nucleoskeleton and cytoskeleton complex; LBR, lamin B receptor; STING, stimulator of interferon genes. Bold text represents higher ion concentration. Created with BioRender.com.

The structure of the NE (**Figure 1**) comprises two functionally distinct lipid membranes, the outer nuclear membrane (ONM) and the inner nuclear membrane (INM), which give rise to a functional compartment between them, known as the perinuclear space (1, 3). The ONM is continuous with the endoplasmic reticulum (ER) making the perinuclear space continuous with the ER lumen (4). The INM can directly interact with proteins within the nucleoplasm, with invaginations of INM reaching deep within the nucleoplasm, forming a nucleoplasmic reticulum (10, 11). An important interaction of the INM proteins is with type V intermediate filament proteins (nuclear lamins) to form the nuclear lamina at the nucleoplasmic surface of the INM (12). Divided into A/C-type and B-type based on their gene sequence, nuclear lamins are important for a number of nuclear functions (6, 13), including nuclear mechanical support and positioning (14, 15), chromatin organisation (16, 17), gene expression (18, 19), nuclear pore complex organisation (20, 21), NE breakdown/repair (22, 23), DNA synthesis (24), intracellular signalling (25–27), cell differentiation, polarisation and programmed cell death (28–30).

The INM and the ONM are only permeable to small non-polar molecules and sustain nucleoplasm membrane potential approximately -15mV with respect to the cytoplasm (31, 32). Polar molecules, ions and macromolecules are able to pass between nucleoplasm and cytoplasm through the nuclear pore complexes (NPCs), which are formed upon INM and ONM fusion (33).

Approximately 1% of human proteins have been experimentally demonstrated to be in the NE to date; and it is known that these proteins, and the complexes they form, add to the complexity of the NE structure and the signalling possibilities (34). The NE-associated proteins that have been the most studied in immune cells are the lamins.

## Lamin Proteins

Lamins A and C, which are created *via* alternative splicing of the *LMNA* gene, are the major components of the nuclear lamina (**Figure 1**). Cellular functions of lamin A/C, include regulation of gene transcription, NPC formation, nuclear positioning and stability; and have been studied in a number of cell types (35). Several studies have demonstrated the important roles of NE lamins in various immune cell functions.

Neutrophils are usually the first cells of the innate immune system that migrate to sites of inflammation; and while they have potent anti-microbial actions, inappropriate accumulation of these active cells can contribute to immune paralysis and organ failure in severe inflammation (36). Low lamin A/C expression in neutrophils appears to contribute to a characteristic nuclear lobulation, flexibility and the ability of these cells to migrate through narrow spaces towards a site of infection (37–39). Interestingly, neutrophil nuclear morphology was reported to be of less significance than NE lamin content for neutrophil mobility (40). Mechanistically, a low lamin A/C NE is thought to reduce the prevalence of lamin-bound chromatin, hence altering chromatin distribution and increasing its mobility within the nucleus, facilitating chromatin flow in the direction of nuclear movement (16, 41, 42). Reduced lamin A/C expression was also suggested to facilitate NE rupture and nuclear protein transport during the formation of neutrophil extracellular traps (NETosis). There are also reports that low lamin A/C levels account for the short lifespan of neutrophils (43). How neutrophils control NE ruptures to facilitate NETosis requires further study.

With regards to the other major cell type of the innate immune system, monocytes/macrophages, it was reported that high levels of lamin A/C expression induced NF-κB nuclear translocation *via* the IKKβ/IκBα/NF-κB pathway and increased the expression of proinflammatory genes, such as *Il6*, *Tnf*, *Ccl2*, and *Nos2*, in adipose tissue macrophages (44). This is in line with the observation that lamin A/C deletion reduced the lipopolysaccharide-induced expression of these genes in macrophages. Importantly, the study established that macrophage lamin A/C levels are linked to adipose tissue inflammation and obesity-induced insulin resistance seen in type two diabetes (44). Lamins A/C levels were also suggested to affect tumour-associated macrophage activity. The c-Fos protein, which influences macrophage responses in cancer (45–47), was shown to interact with and be regulated by lamin A/C (25). Another study demonstrates that lamin A/C gene silencing reduces the number of Leishmania parasites per sampled macrophage and reduces the percentage of infected macrophages in Leishmaniasis (48). Generally, macrophages express higher levels of lamin A/C compared to some other immune cells, which may contribute to macrophages’ proinflammatory actions and comparably longer life span than other innate immune cell types (49). The level of lamin A/C expression in dendritic cells was reported to fall between that of macrophages and neutrophils (6, 50). The presence of functional lamin A/C in mature dendritic cells was demonstrated to limit the nuclear egress of viral capsids and the spread of infection in the case of herpes simplex virus type 1 (51). The role of lamins in dendritic cells, however, needs further investigation.

In the case of cells of the adaptive immune system, T lymphocytes transiently increase their lamin A/C expression upon antigen presentation, with none or weak lamin A/C expression being detected in resting human or mouse T and B lymphocytes (52–54). This demonstrates that NE protein composition can change depending on the cellular activation state. The T lymphocytes’ transient increase in lamin A/C expression was suggested to accelerate the immunological synapse formation between T lymphocytes and antigen presenting cells. Another possible explanation suggested was that transient increase in lamin A/C is related to the lymphocytes’ need to migrate out of lymph nodes and into target tissues (55). Interestingly, the role of lamin A/C may be more important in lymphocyte differentiation during infectious and autoimmune responses (56, 57). Lamin A/C absence reduces T helper 1 lymphocyte differentiation, but does not affect T helper 2 differentiation; this is consistent with significantly weakened lymphocyte responses to both viral and intracellular parasite infections seen in lamin A/C deficiency (58). In comparison, lamin A/C absence increased regulatory T lymphocyte differentiation, reduced T helper 1 differentiation and was protective in a model of inflammatory bowel disease (59, 60). However, exactly how T- lymphocytes regulate their lamin A/C expression is not clear with possible mechanisms including the AKT/protein kinase B signalling pathway (61, 62), microRNA (63) and/or retinoic acid (39, 64) being proposed.

In addition to lamin A/C, lamin B1 has been demonstrated to regulate lymphocyte somatic hypermutations and lymphoid malignancy progression (65). The loss of lamin B1 has also been suggested to contribute to increased inflammation seen with aging (66, 67), although the relative contribution of the innate immune cells in this observation requires further investigation. The Lamin B receptor (LBR) (**Figure 1**) appears critical for nuclear lobulation, rapid migration and normal function of neutrophils (68). Emerin, an INM protein (**Figure 1**), regulates nuclear polarity, a crucial feature of migratory cells (69). Finally, a pool of stimulator of interferon genes (STING), a DNA sensor and adaptor protein, was recently confirmed in the NE (70), which will be discussed later.

## NPC and LINC Complexes

After the lamins the most well studied protein assemblies in the NE are the nuclear pore complexes (NPCs). The NPCs are multi-protein complexes, each comprising around 500 to 1000 individual protein molecules, of which the main structural proteins are termed nucleoporins (71, 72). There are around 40 different nucleoporins with their nomenclature being mostly based on their mass (73). The central channel of the NPC is located at sites of fused INM and ONM and is lined by nucleoporins. One of the most well-researched functions of NPCs is to control the movement of polar molecules and macromolecules across the NE. NPCs are also involved in chromatin organization, regulation of gene expression and DNA repair (33, 74).

Several studies have addressed the role of individual nucleoporins, and therefore by implication NPCs, in immune cells. Faria et al. (75) demonstrated that lower than normal levels of nucleoporin 96, a core element which is essential for the NPC assembly (71), resulted in altered immune responses, decreased interferon-mediated expression of major histocompatibility complexes, impaired antigen presentation by antigen presenting cells, which consequently reduced T lymphocyte proliferation and increased susceptibility to infection (75). Two other nucleoporins, nucleoporin 88 and nucleoporin 214, were shown to contribute to appropriate nuclear accumulation of some key immune transcription factors including NF-κB, thus regulating the relative strength and duration of immune responses at the level of the NPC in Drosophila and mammalian cells (76–78). Finally, nucleoporin 210 is critical for the survival of circulating T lymphocytes, regulates T cell receptor signalling and hence, adaptive immunity (79).

One important interaction of NPCs is with linker of nucleoskeleton and cytoskeleton (LINC) complexes. Evidence suggests (80–85) that this interaction couples the cytoskeleton and the nucleoskeleton, allowing cytoskeleton rearrangements activated by mechanical stimuli to regulate nucleocytoplasmic transport. LINC complexes comprise SUN proteins on the INM and KASH/Nesprin proteins on the ONM, which interact with each other across the perinuclear space (**Figure 1**). Mechanical stimuli have been demonstrated to propagate *via* a direct pathway from cell membrane integrins to SUN proteins on the NE, where SUN proteins transmit the signals *via* mechanical connections to NPCs, lamins and chromatin (80–83). The transmission of mechanical stimuli significantly affects the NPC conformation *via* the Nup153-SUN1 connection, the nucleocytoplasmic transport through NPCs and the state of chromatin packing (84). Importantly, different studies show that NPC and LINC complexes coexist on the NE and have a number of interdependent cellular functions. Firstly, LINC complexes regulate NPC assembly and distribution in the NE. SUN1 was shown to colocalize with NPCs and was suggested to reduce the spacing between the INM and the ONM, thus promoting membrane fusion at sites of NPC assembly (86–88). NPCs control the trafficking of SUN proteins across the NE for INM localization. For example, human SUN2 possesses a classical nuclear localization signal, that binds to the importin-alpha-importin-beta heterodimer and mediates transport through the NPC (89). There are also reports that LINC complexes can affect nucleocytoplasmic transport through NPCs, and a study by Li and Noegel (90) reported that SUN1, *via* interaction with nucleoporin 153, regulates efficient nuclear export of mRNA in mammalian cells (90).

While the significance of LINC complexes in immune cells is yet to be fully investigated, SUN2 regulates T lymphocyte proliferation, function and viability (91). In neutrophils, LINC expression along with lamin and LBR relative expression influence cell migration, which is critical for neutrophil function (92). With increasing recognition that mechanical stimuli can impact on immune cell function, see the following sections, the relationship between NPC-LINC and immune cell activation profiles merits further investigation.

## NE Ion Channels

The vast majority of studies investigating ion channels have focused on ion channels in plasma membranes and intracellular double membrane systems of mitochondria and ER. In comparison, the double membranes of the NE have received relatively little attention. Nevertheless, the INM and ONM do contain a range of ion channels (**Figure 1**) (34, 93–95).

The lack of permeability of NE membranes to ions together with the presence of ion channels and transporters in the NE allow ionic gradients across INMs and ONMs to be generated and maintained irrespective of the NPCs’ activity. Various genetic, pharmacological and electrophysiological approaches have identified a number of ion channels present on INMs and ONMs of different cell types, from liver to immune cells. These include calcium ion channels which could contribute to the regulation of gene transcription, cell cycle control and other nuclear processes (34, 96), potassium ion channels which possibly regulate other NE voltage-sensitive channels by setting the membrane potential (32, 94, 97, 98) and chloride ion channels, potentially participating in osmotic volume regulation of the nucleus (32, 34, 98).

Considering the NE of innate immune system cells, we have recently reported the expression of large conductance (~100-300pS) voltage and calcium activated potassium ion channels (BK channels; K_Ca_1.1) (94). Importantly this study showed that nuclear BK channels regulate cAMP response element binding protein (CREB) phosphorylation in RAW264.7 macrophages. CREB is an important transcription factor with major roles in immune cells (99–101). Moreover, the study reported that nuclear calcium and calmodulin dependent kinases II and IV were involved in regulation of CREB by nuclear BK channels in macrophages (94). Similar findings were previously reported in hippocampal neurones (102). Based on knowledge from other cell types, one can hypothesise that blockade of nuclear BK channels in macrophages influences the activity of other nuclear ion channels or the Ca^2+^ filling state of the perinuclear space, possibly changing the NE membrane potential. This eventually could lead to increased nuclear calcium, activation of calcium-dependent kinases and, finally, activation of various transcription factors, including CREB. BK channels have also been identified on the NE of microglia, which are the resident macrophage-like cells of the central nervous system (103). In this study it was hypothesised that NE BK channels are involved in microglia response to stimuli, facilitating nitric oxide and cytokine production, potentially by regulating calcium and potassium fluxes in the nucleus (103).

To date only a few papers have addressed the role of NE ion channels in lymphocytes. Franco-Obregon et al. (95) reported that chloride channels (105pS) predominated on the ONM of T lymphocytes, while channels on the ONM of B lymphocytes were primarily cation selective (52pS) (95). Although only anion channels were detected in T lymphocyte ONM, both anion and cation channels were observed in B lymphocyte ONM (95). Generally, INMs are considered to have a different ion channel composition compared to ONMs (95, 104). On the INM of T-lymphocytes the presence of both large conductance anion selective channels (370pS) and cation selective (152pS) channels was reported (93). However further studies are required to fully characterise the ion channels present in the NE of lymphocytes. It will be interesting to see if NE ion channels in lymphocytes regulate aspects of T-lymphocyte physiology such as gene transcription, proliferation, differentiation and apoptosis.

Evidence is now beginning to accumulate that suggests NE ion channels are important in immune cell functions and activation. However, this is a complex system with ion channel type, location (INM or OMN), and subunit composition all impacting the functional significance of the NE ion channels.

## Perinuclear Ion Store

As already mentioned, the two membranes of the NE form a lumen, referred to as the perinuclear space (**Figure 1**). The selective permeability of INM and ONM to ions contributes to ion concentration gradients across the two membranes and allows the perinuclear space to act as a cellular ion store. It is not unreasonable to assume that changes in ion concentration within the perinuclear space might have significant effects on nuclear/cellular functions. The idea that the nucleus possesses an autonomous calcium store and signalling system, which generates its own calcium transients independent of cytoplasmic calcium changes, has been debated for years. Supporting this idea several studies suggest that nuclear calcium is independent of cytoplasmic calcium and NPC-mediated calcium transport (102, 105–109). In liver cells, Leite et al. (105) showed that upon ATP stimulation nucleoplasmic calcium signalling preceded the cytoplasmic calcium signal, thus leading to the suggestion of an autonomous nuclear calcium signalling system (105). Similarly, in hippocampal neurones, Li et al. (102) reported that nuclear BK channel block induced nucleoplasmic calcium elevation without changing cytoplasmic calcium concentration (102). Other studies identified the nucleoplasmic reticulum, formed from INM invaginations, as a nuclear calcium store and regulator. The presence of calcium storage proteins, calreticulins (110), calcium releasing inositol-1,4,5-trisphosphate receptors (105) and calcium pumps (111, 112) in the NE and its invaginations further supports the idea of a fairly autonomous NE calcium store (107, 108). There are also calcium-mobilizing ryanodine receptors in lymphocyte nuclei, while in monocytes and neutrophils their localization is reported to be extranuclear (113). Perinuclear calcium, when released into the nucleoplasm, is thought to regulate nuclear calcium-sensitive gene expression through regulation of calcium-dependent enzymes and transcription factors (94, 102). In relation to cells of the immune system, nuclear calcium was found to control T lymphocyte fate decision between active proliferation and a hyporesponsive state (114). Moreover, it has been debated if the Ca^2+^ filling state of the perinuclear space influences NPC size and permeability. Several contradictory reports exist. Some show that NE calcium depletion significantly increases the proportion of NPCs blocked by a central plug and attenuates the nuclear influx of proteins, while others argue that depletion of calcium from the NE lumen does not significantly alter the movement of the green fluorescent protein across the NE (115–122).

## Is the NE an Integrator of Mechanical and Chemical Signalling Pathways in Inflammation?

It is now apparent that along with the diverse array of extracellular chemical signals that immune cells respond to, such as pathogen-associated molecular patterns (PAMPs) and damage-associated molecular patterns (DAMPs), these cells can also respond to their physical environment. This mechanosensing may be of particular relevance during an inflammatory response where the leukocytes of the innate immune system need to essentially “travel and deform” to perform their function. During inflammation leukocytes can be subjected to changes in mechanical and compressive forces, such as those experienced during cell migration and cell extravasation; osmotic forces, which can occur during cell swelling and tissue damage; and forces associated with phagocytosis such as deformation of the leukocytes and engagement with targets of different shape and orientation. Tissue composition such as extracellular matrix stiffness, hydrostatic pressure and cell crowding can also provide mechanical signals to leukocytes. A complete map of the cellular pathways activated by mechanosensing in immune cells is yet to be fully elucidated but appears to involve plasma membrane ion channels such as PIEZO1 (123) and TRPV4 (124), adhesion molecules and cytoskeleton rearrangement, and the reader is pointed towards several recent reviews on the subject (125–127). The NE and its associated proteins are also involved in the signal transduction mechanisms activated by changes in mechanical forces through interactions between the cytoskeleton, LINC protein complexes and the lamin network which attaches the INM to lamin-associated-domains (LADs) within chromatin and thus influences chromatin dynamics (128, 129). Along with evidence that the NE represents a core component of mechanical signal transduction, a number of seminal studies have now also demonstrated that the NE itself is a sensor of mechanical forces (130–132), a function it may be well suited for (133). However, to date only a few reports have investigated the role of NE components in immune cells in response to mechanical forces (134).

As discussed above, the NE-associated proteins have effects on the cellular functions of immune cells and the NE is involved in the transduction pathways of many mechanical stimuli encountered by immune cells during an inflammatory response. In addition, the NE contains and interacts with a variety of structural proteins, ion channels, pore proteins and transporters, which suggests the NE may be important in cell signalling cascades which regulate nuclear functions such as heterochromatin formation, gene expression, nuclear morphology and nuclear repair. For example, ion channels within the NE could regulate Ca^2+^ levels in the perinuclear space which can regulate gene transcription (135), see the above section. But is there evidence that mechanosensing by NE can regulate cell signalling cascades and, therefore, immune cell function during an inflammatory event?

Yes-associated protein (YAP) is a transcription cofactor that regulates macrophage innate inflammatory pathways (136). Importantly, YAP activation and translocation into nucleus has been demonstrated to be regulated by NE flattening and nuclear pore opening caused by mechanical forces and extracellular matrix (ECM) stiffness (137, 138). The NE protein Emerin, which appears to be an important component in mechanotransduction pathways in the NE (139), binds and activates histone deacetylase 3 (HDAC3) (140), an important regulator of autoimmunity and inflammatory gene expression (141, 142). Finally, osmotic cell swelling, which causes increased NE tension, results in cPLA2 localisation to the INM of zebrafish leukocytes. This mechanism in the dying cells appeared to facilitate the mobilization of other leukocytes to clear necrotic debris, presumably by the release of eicosanoids (134). It will be interesting if these final observations can be extended to mammalian leukocytes and lymphocytes.

An important emerging principal is that the magnitude of NE perturbations will determine the cellular pathways activated (143), with the most severe mechanical stressors, or possibly a milder mechanical stressor but in a permissive cellular environment, leading to NE rupture. This does not necessarily lead to cell death as NE ruptures can be repaired. However, NE ruptures can cause the mixing of material from the nucleoplasm with the cytoplasm and this may be an important mechanism of leukocyte activation. The DNA sensing cGAS-STING and AIM-2 pathways are part of the innate immune response to viruses, whereby cells are activated by inappropriate cellular localisation of viral DNA which results in the production of type I interferons (144). However, these pathways can be activated by endogenous DNA spilling from the nucleus, and this may contribute to inflammatory diseases including Aicardi-Goutières Syndrome (145–148). NE rupture may also contribute to the low-grade chronic inflammation associated with old age, referred to as inflammaging. NE instability increases in senescent cells and is believed to contribute to the establishment of the senescence-associated secretory phenotype (SASP) (149, 150). SASP, which is also associated with ER stress, is proposed to be one of the main contributors to inflammaging (151). It will be important to investigate the effects of NE perturbations caused by mechanical stimuli in inflammatory environments. Taken together, it would appear that the NE is important in multiple immune functions of leukocytes.

## Future Prospective

When studying the role of the NE in immune cells a number of considerations need to be taken into account. As well as studying the NE in the setting of the whole cell it is also possible to carry out studies on isolated nuclei, NE preparations and reconstituted systems involving nuclei being placed is cytosolic preparations (94, 152, 153). The choice of experimental system employed will depend on the cellular process to be investigated and when using isolated nuclei or NE it is important to check for sample contamination with membrane material from other organelles.

Most modern cellular and biochemical techniques, for example immunofluorescence, Western blot analysis and electrophysiology, can be applied directly to isolated nuclei and NE, although determining if a protein is located within the inner or outer nuclear membrane still requires careful consideration. While nuclei isolation gives a direct access to the NE, it limits our understanding of how nuclei and the NE integrate within a functioning cell. Therefore, there is a need to develop methodologies which specifically target proteins in the nuclei and NE when investigating in whole cells. The reason for this is that many proteins in the nuclei and NE are not exclusive to these sites and can be found in other organelles and membranes within the cell. Most current pharmacological and genetic tools used to investigate protein function will not discriminate between the same protein found at different cellular locations, thus attributing specific cellular functions to NE located proteins is problematic. It will be important to develop pharmacological and genetic tools which specifically target the constituents of the nuclear membrane, both to facilitate basic research and hopefully future therapeutic development.

Considering the role of the NE in immune cells outstanding questions to be addressed are:

What are the differences and changes in NE composition between immune cell types and their various activation states?

Can selective targeting of NE components be achieved in a functioning immune cell and therefore help elucidate the role of these components in immune cells?

Can NE proteins influence gene transcription in immune cells, either by regulating transcription factor activation or by epigenetic mechanisms? And is this specific to certain genes?

Do NE components represent possible therapeutic targets for the treatment of autoimmune and inflammatory diseases?

## Conclusion

The nucleus is sometimes referred to as “a cell within a cell”, and one would expect such an emotive phrase to be the focus of much research. It is surprising that the membrane which delineates this structure has received relatively little research attention. This may stem from the traditional view that the NE plays only a minor role in cellular physiology. One aim of this review is to increase the awareness of the roles that the NE may play in the cell and in particular in immune cell function.

The NE, through its various components such as lamins, NPC and LINC complexes, cation and anion channels has been shown to act as a regulator of immune cell function summarised in **Figure 1**. In the immune system the NE’s influences range from macrophage polarization and lymphocyte differentiation to cell migration and lifespan (**Figure 1**). In addition, the NE can also act as a sensor of intracellular and extracellular environments and physical stresses on the cell. However, the investigation of the NE in immune cells is still in its infancy. Today mitochondria and the ER are considered crucially important organelles in shaping the actions of cells of the immune system. Time will tell if the NE and its associated structures become the 3^rd^ member of this trinity.

## Author Contributions

All authors listed have made a substantial, direct, and intellectual contribution to the work and approved it for publication.

## Conflict of Interest

The authors declare that the research was conducted in the absence of any commercial or financial relationships that could be construed as a potential conflict of interest.

## Publisher’s Note

All claims expressed in this article are solely those of the authors and do not necessarily represent those of their affiliated organizations, or those of the publisher, the editors and the reviewers. Any product that may be evaluated in this article, or claim that may be made by its manufacturer, is not guaranteed or endorsed by the publisher.
